# Do Challenge Stress and Hindrance Stress Affect Quality of Health Care? Empirical Evidence from China

**DOI:** 10.3390/ijerph15081628

**Published:** 2018-08-01

**Authors:** Tengyang Ma, Tianan Yang, Yilun Guo, Yifei Wang, Jianwei Deng

**Affiliations:** 1 School of Management and Economics, Beijing Institute of Technology, Beijing 100081, China; 18829285968@163.com (T.M.); tianan.yang@bit.edu.cn (T.Y.); 18810776591@163.com (Y.G.); 2 Sustainable Development Research Institute for Economy and Society of Beijing, Beijing 100081, China; 3 School of Humanities, Beijing Institute of Technology, Beijing 100081, China; 18811603825@163.com

**Keywords:** challenge stress, hindrance stress, health, quality of health care, Chirurgisches Qualitätssiegel

## Abstract

Severe job stress has adverse effects on the health of Chinese healthcare workers. We investigated associations between job stress, health, and quality of health care among Chinese healthcare workers. To analyze associations between stress, health, and quality of health care among healthcare workers in 74 Chinese hospitals, we surveyed 2426 healthcare workers of primary, secondary, and tertiary hospitals in Western, Central, and Eastern China in 2017. Structural equation modelling was used to examine relationships between job stress, health, and quality of health care. The mediating effect of health on the association between job stress and quality of health care was examined with the Sobel test. In the final model, health had a moderate direct positive effect on the quality of health care (β = 0.24; *p* < 0.001). Challenge stress had a direct inverse effect on health (β = −0.05; *p* < 0.05) and a significant direct positive effect on the quality of health care (β = 0.26; *p* < 0.001). Hindrance stress had a significant inverse effect on health (β = −0.37; *p* < 0.001) and a moderate inverse effect on the quality of health care (β = −0.19; *p* < 0.001). The correlation between challenge stress and hindrance stress was significant and positive (β = 0.59; *p* < 0.001). A partial mediation effect was in the final model. The health status of healthcare workers is an important concern at all levels of Chinese hospitals. To improve quality of healthcare, appropriate challenge stress is recommended among young staff, and interventions targeting hindrance stress should be developed and implemented in all hospital departments.

## 1. Introduction

Quality of health care is assessed mainly in relation to inputs, processes, and outputs [1,2]. Popular assessment tools include the ISO9002 standard (comprising of hospital equipment, human resources, and overall management assessment)and the SERVQUAL scale of patient satisfaction [3]. These instruments mainly focus on hospitals and patients but ignore healthcare workers. However, increasing the overall health of healthcare workers might improve the quality of health care by reducing medical errors [4,5] and improving patient care [6,7]. Since the quality of health care cannot be assessed by only macro statistics or third-party evaluations [8], Chirurgisches Qualitätssiegel (CQS) [7,8] was developed to measure quality of health care through self-evaluation of healthcare workers.

The health of healthcare workers and the quality of the health care they provide are severely impaired by job stress [8,9]; thus, it is urgent to improve the quality of health care in this population [8,10]. The main concern regarding job stress is its adverse effects on the health and performance of healthcare workers. Most previous studies of job stress did not differentiate between challenge stress (job stress that benefits career development, such as shift positions, job responsibility, and workload) and hindrance stress (job stress perceived as unbearable, such as burdensome work policies, conflict with others, and job insecurity) [9,11]. Researchers have focused on the direct effects in analyses of job stress, health, and the quality of health care but have ignored the possible mediating effect of health, although it is obvious that healthcare workers cannot perform well when in poor health.

**Figure 1:**
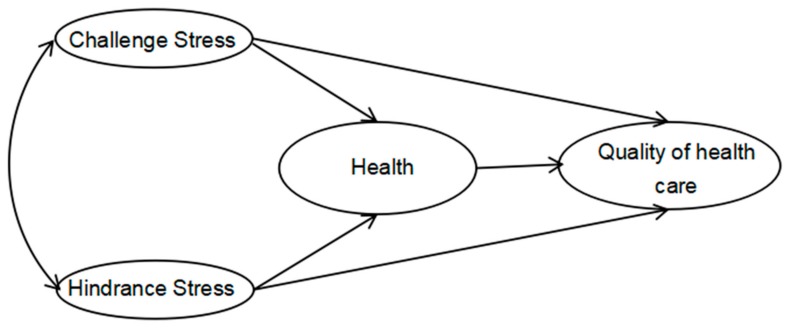
Proposed model of how challenge stress, hindrance stress, and health affect the quality of health care.

Robust empirical studies showed that Chinese healthcare workers are exposed to considerable medical violence [12,13,14], and the occupational exposure for healthcare workers in China was worse than that of their global colleagues [15]. This might cause more mental illness and post-traumatic stress disorder (PTSD) [12,14,16]. But at the same time, Health China 2030 Plan calls for continuing enhancement of quality of health care [17]. Therefore, in this supply-side analysis, we examined the effects of challenge stress and hindrance stress on the quality of health care among Chinese healthcare workers. Figure 1 shows a model of the study hypothesis.

## 2. Methods

### 2.1. Sample

To investigate the relationships between job stress, health, and the quality of health care among healthcare workers in China, we surveyed doctors, nurses, technical staff, and administrative staff in 2017, after receiving ethics approval. We distributed 2590 questionnaires, and 2426 healthcare workers at all levels of Chinese hospitals nationwide (Western, Central, and Eastern China) answered and were included in the final sample (response rate, 93.7%). Chinese hospitals are designated as primary, secondary, and tertiary hospitals in accordance with their function, facilities, and technical capabilities [18]. Tertiary hospitals provide high-level specialized medical and health services in several regions and conduct higher education and scientific research. They are usually comprehensive or general hospitals at the city, provincial, or national level and have more than 500 beds. Primary and secondary hospitals mostly provide services to one or more smaller communities. Due to substantial regional differences and heterogeneity, we randomly selected primary, secondary, and tertiary hospitals from Western, Central, and Eastern China.

For each target hospital, we used unique employee identification numbers to select 5% to 12% of healthcare workers. This ensured data objectivity and integrity. Ultimately, data from 2426 questionnaires were analyzed. All instruments used are reliable and validated in China [19].

### 2.2. Variables and Instruments

The quality of health care was assessed by using the Chirurgisches Qualitätssiegel (CQS); a short version (13 items, Cronbach α = 0.959) of a German self-assessment instrument that was reported to be a reliable and robust indicator of healthcare worker performance [20,21]. The CQS was developed in accordance with the Canadian Physician Achievement Review [8] and uses a 5-point Likert scale ranging from “very good” to “bad”. The three dimensions, shown in Table 1, are psychosocial care, diagnosis/therapy, and quality assurance. Chirurgisches Qualitätssiegel (CQS) has high reliability and acceptable psychometric properties. Higher values represent better quality of health care.

Job stress was measured with the 11-item Challenge and Hindrance-related Self-Reported Stress (C-HSS) scale, see Table 1. A five-point Likert scale (1 = no stress; 5 = greatest stress) is used to evaluate challenge stress and hindrance stress. Greater job stress is reflected by higher values (Cronbach α = 0.87 − 0.75) [11].

Health was measured by an 8-item Short-Form Health Survey (SF-8 general health: six-point Likert scale, 1 = excellent, 6 = very poor; remaining items: five-point Likert scale, 1 = not at all, 5 = could not do daily activities; Cronbach α = 0.910) [22]. This instrument analyzes the same eight health domains as the Short-Form Health Survey 36 (SF-36) [22,23,24,25]. For example, the item “Overall, how would you rate your health during the past four weeks?” asks respondents to evaluate their health status. The scoring was reversed for these items so that higher values reflect better health status.

### 2.3. Statistical Analysis

SPSS 21.0 (IBM Corp., Armonk, NY, USA) and AMOS 21.0 (IBM Corp.) were used to prepare and analyze the data unless otherwise noted. Statistical Analysis comprised of descriptive analysis, correlation analysis, path analysis, subgroup analysis, and structural equation modeling (SEM). We used structural equation modeling to examine and analyze relationships between challenge stress, hindrance stress, health, and quality of health care.

In SEM, quality of health care, challenge stress, hindrance stress, and health were the four latent variables constructed by using the Chirurgisches Qualitätssiegel (CQS) indicators, the C-HSS and 8-item Short-Form Health Survey indicators. Pearson correlation analysis was used to determine the significance of correlations between challenge and hindrance stress, health, and the quality of health care before imputing the indicators into the SEM. The criteria used to evaluate good global fit were; a root mean square error of approximation less than 0.08; and goodness-of-fit index, normed fit index, comparative fit index, and Tucker–Lewis index values of 0.90 or higher [26]. These indicators were used to assess model fit. The Sobel test was used to examine the effect of the mediator.

We used standardized regression coefficients (β) in SEM to express complex effect relationships among variables (challenge stress, hindrance stress, health, and quality of health care). To determine if β differed by subgroup, we conducted subgroup analyses of respondents classified by sex, age, and hospital type.

## 3. Results

### 3.1. Demographic Characteristics of Respondents

The demographic characteristics of the respondents are shown in Table 2. Among the 2426 respondents detailed in Table 2, 36% were male. Most healthcare workers (76%) were younger than 40 years: 9.4% were younger than 25 years, 28.4% were 25–30 years of age, 23.8% were 31–35 years of age, and 14.4% were 36–40 years of age. Most respondents had a bachelor’s degree (46.1%) and had a job title of entry-level (45.6%). The respondent seniority (years of employment) was evenly distributed, and the largest group had a seniority of 6–10 years (25.9%). A plurality of respondents (24.7%) worked in the internal medicine department. The mean values for the quality of health care, challenge stress, hindrance stress, and health items, see Table 1, were moderate and varied little.

### 3.2. Correlations between Job Stress, Health, and the Quality of Health Care

Correlation analysis (*r*) showed positive correlations between items within the same construct, as shown in Table 3. Health was significantly inversely correlated with challenge stress (*r* = −0.25) and hindrance stress (*r* = −0.36) and significantly positively correlated with quality of health care (*r* = 0.25). Challenge stress was significantly positively correlated with hindrance stress (*r* = 0.49) and had a positive impact on the quality of health care (*r* = 0.08). The quality of health care and hindrance stress were significantly inversely correlated (*r* = −0.14).

### 3.3. SEM

**Figure 2:**
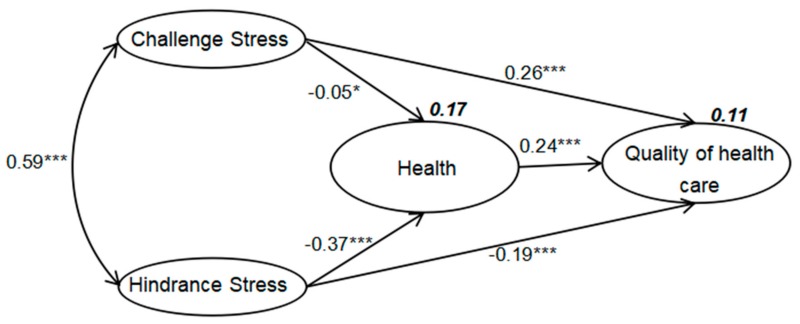
Final structural equation model, with standardized maximum likelihood estimates (Chi square, 1894.033; *p* = 0.000; root mean square error of approximation, 0.060; goodness-of-fit index, 0.931; normed fit index, 0.945; comparative fit index, 0.951; *** *p* < 0.001; * *p* < 0.05).

We used SEM to test the proposed model. In the final structural equation model with standardized maximum likelihood estimates, the criteria for fitness (goodness-of-fit index, comparative fit index, root mean square error of approximation, and normed fit index) indicated that the final model was appropriate. Root mean square error of approximation was 0.060, the value for the goodness-of-fit index was 0.912, the comparative fit index was 0.951, and the normed fit index was 0.945, as shown in Figure 2.

In the final model, the relationships of the four latent variables were examined. Health had a moderate direct positive effect on quality of health care (β = 0.24; * *p* < 0.001). Challenge stress had a direct inverse effect on health (β = −0.05; * *p* < 0.05) and a significant direct positive effect on the quality of health care (β = 0.26; * *p* < 0.001). Hindrance stress had a significant inverse effect on health (β = −0.37; * *p* < 0.001) and a moderate inverse effect on the quality of health care (β = −0.19; * *p* < 0.001). The correlation between challenge stress and hindrance stress was significant and positive (β = 0.59; * *p* < 0.001). Challenge stress and hindrance stress explained 17% of the variability in health, and challenge stress, hindrance stress, and health explained 11% of the variability in the quality of health care, see Figure 2.

After comparing different effects of health, we noted significant indirect effects between challenge stress and the quality of health care (Sobel z = −9.241; *p* < 0.001) and between hindrance stress and the quality of health care (Sobel z = −8.756; *p* < 0.001), which were significantly mediated by health. It was also found that partial mediation of health was between challenge stress and the quality of health care (*p* < 0.001), as well as between hindrance stress and the quality of health care (*p* < 0.01).

Subgroup analysis, shown in Table 4, revealed differences in the effects of challenge and hindrance stress on the quality of health care and health. Among women, workers older than 30 years, and workers at primary hospitals, challenge stress had a significant negative effect on health. However, challenge stress did not have a significant negative effect on health among workers in secondary and tertiary hospitals. All other results of subgroup analyses were identical to those of our final model.

## 4. Discussion

Our analysis yielded three valuable findings. First, challenge stress had a significant positive impact on the quality of health care. Second, health was a robust mediator of challenge stress, hindrance stress, and the quality of health care. Finally, challenge stress had a greater adverse effect in primary hospitals than in secondary and tertiary hospitals. These findings suggest intriguing avenues for future research on the complicated differences between primary, secondary, and tertiary hospitals in China.

Challenge and hindrance stress affect the health of healthcare workers and the quality of health care in distinct ways. The effect of challenge stress on the health of healthcare workers and the quality of health care was significantly negative and positive, respectively, because people usually choose to challenge themselves in order to fulfill their desire for self-development [9,11]. Thus, they improved quality of health care by improving their competence and self-autonomy, as posited by Maslow’s theory and self-determination theory [27,28,29]. These findings and those of subgroup analysis are consistent with previously reported results [8,10]. Subgroup analysis showed that challenge stress had a significant negative effect on health in primary hospitals but not in secondary or tertiary hospitals. Due to the comprehensive reform of public hospitals in China, the process of hierarchical diagnosis now directs more people to primary hospitals for treatment of minor illnesses. Limited evidence suggests that the numbers of inpatients and outpatients have increased in primary, but not in secondary or tertiary, hospitals. Primary hospitals should therefore promote financial awards and social support to healthcare workers to reduce the adverse effects of challenge stress on their health. As even challenge stress has negative health effects, all Chinese hospitals should communicate closely with healthcare workers when assigning tasks and responsibilities during medical treatment to minimize these adverse effects. Furthermore, evidence from our previous report indicates that hospitals need to be aware of the importance of the effects of challenge stress when attempting to improve quality of health care in their hospitals. The job demands–resources model (JD-R model) [30] suggests that hospitals should provide opportunities to participate in career development training, advanced seminars, and financial and social support, along with increased work responsibilities.

Hindrance stress significantly affected the health of healthcare workers and the quality of the health care they provided. Cognitive theory indicates that negative emotions damage workers’ physical and mental well-being, thus hindering their status, work, and performance [31]. The present subgroup analysis showed that hindrance stress had negative effects on health and the quality of health care in primary, secondary, and, particularly, tertiary hospitals. This result is consistent with those of previous studies, which showed that job stress had an adverse effect on health and reduced the quality of health care, especially in Chinese tertiary A hospitals [6,10,11,12,16]. Hospitals at all levels need to focus on reducing hindrance stress and alleviating its adverse impact by providing job resources that help workers cope with work hindrances. Cutting down on red tape, reforming the rotational system, and promoting job security by providing supervisor support and coworker support are particularly important in hospitals.

Health had a significant effect on the quality of health care because it is an inherent factor in the work commitment of healthcare employees [32]. Protection motivation theory (PMT) states that healthcare workers provide better health care when they are in good health, especially if they understand health-related behaviors and have undergone health education [32,33]. We previously reported that health was a mediator between challenge stress, hindrance stress, and the quality of health care. Healthcare workers in poor health may fail to provide high-quality health care and may even compromise the healthcare system [10]. Relevant agencies and departments should acknowledge the importance of health, establish health assessment tools that track the health status of hospital workers (e.g., by using standardized questionnaires to measure department performance), and intervene when necessary.

Job stress is a particular concern for women and young workers in all types of hospitals. Challenge stress had a significant negative effect on women’s health because women are more emotional and sensitive to stress than men when their work-family relationship and workload are out of balance [34]. Hospitals should provide more humanistic care, timely counseling, and interventions supporting the physical and mental health of female workers. Hindrance stress had a strong negative effect on health and quality of health care among younger workers because of their limited work experience or an inappropriate path for their career development [35,36]. Health and quality of health care were worse in this subgroup than in older workers. Therefore, hospitals need to provide organizational support, create a harmonious organizational atmosphere, and ease hindrance stress among young employees.

The path of job stress to quality of health care must be improved. The present study suggests that both variables have a direct role, and no mediator of job stress and quality of health care has been identified [8,10]. Existing evidence shows that health (i.e., the entire range of physical, mental, and social functioning, as defined by the World Health Organization) affects the quality of health care [9,25,37]. Job stress causes anxiety, depression, and even mental illness among healthcare workers [38,39]. In addition, healthcare workers have high prevalences of insomnia, poor disease resistance, and cancer [8,40]. Poor health diminishes quality of health care [10,37]. This study introduced health as a mediator, thus clarifying the research path of challenge stress, hindrance stress, and the quality of health care.

This study suggests potential measures of quality of health care, which can be assessed from the perspective of patients or that of healthcare workers [9]. We evaluated the patient perspective on quality of health care with the SERVQUAL scale, which analyzes functional quality (such as the attitude of healthcare workers) of care delivered to the patient [9,41]. However, the SERVQUAL scale cannot assess the technical quality of health care (technical accuracy of diagnoses and procedures) [41,42]. We used the self-reported CQS to evaluate the perspective of healthcare workers. Cognitive theory maintains that healthcare worker performance is enhanced when they evaluate systemic shortcomings [27]. In addition, attribution theory suggests that, because healthcare workers are the direct providers of health care, their wellness (physical, mental, and social) determines the output of health care from their instinct motivation [43]. This study suggests new measures of quality of health care (self-evaluation of healthcare workers), encourages consideration of the wellness of healthcare workers as a means to improve quality of health care, and, most importantly, provides robust empirical evidence that shifts the focus of quality in healthcare reform from the demand side to the supply side.

## 5. Limitations

First, although the data collected at primary, secondary, and tertiary hospitals are representative, future studies should collect larger samples, to allow for a more comprehensive comparison of the hospital types in China. Second, use of a self-assessment scale for healthcare workers is complicated by the fact that workers have different intrinsic evaluation criteria. Future studies should use objective data in order to validate our subjective data. Finally, our cross-sectional design limits the generalizability of our findings.

## 6. Conclusions

The severe job stress and poor work environment of Chinese healthcare workers greatly increase their work burden in comparison with other occupations. Although hindrance stress was associated with poor worker health and quality of health care, challenge stress improved quality of health care. The use of a self-assessment instrument is a promising method to measure supply-side quality of health care among healthcare workers. To improve quality of health care, researchers and policymakers should shift their focus from investment in hospitals, international standard classification, and patient satisfaction to the wellness of healthcare workers, that is, from the demand side to the supply side of quality of health care.

## Figures and Tables

**Table 1:** Mean and standard deviation (SD) of the items of challenge stress, hindrance stress, health, and the quality of health care.

	Item	Mean	SD
Challenge Stress (CS) (1–6)	CS 1. The number of projects and/or assignments I have.	3.47	0.85
	CS 2. The amount of time I spend at work.	3.51	0.84
	CS 3. The volume of work that must be accomplished in the allotted time.	3.40	0.91
	CS 4. Time pressures I experience.	3.45	0.90
	CS 5. The amount of responsibility I have.	3.58	0.90
	CS 6. The scope of responsibility my position entails.	3.47	0.88
Hindrance Stress (HS) (1–5)	HS 1. The degree to which politics rather than performance affects organizational decisions.	2.82	1.12
	HS 2. The inability to clearly understand what is expected of me on the job.	2.33	1.06
	HS 3. The amount of red tape I need to go through to get my job done.	3.07	1.05
	HS 4. The lack of job security I have.	3.05	1.15
	HS 5. The degree to which my career seems stalled.	2.99	1.06
Health (H) (1–8)	H 1. Overall, how would you rate your health during the past 4 weeks?	3.39	0.92
	H 2. During the past 4 weeks, how much did physical health problems limit your physical activities (such as walking or climbing stairs)?	3.79	0.95
	H 3. During the past 4 weeks, how much difficulty did you have doing your daily work, both at home and away from home, because of your physical health?	3.81	0.94
	H 4. How much bodily pain have you had during the past 4 weeks?	4.16	1.20
	H 5. During the past 4 weeks, how much energy did you have?	3.38	0.91
	H 6. During the past 4 weeks, how much did your physical health or emotional problems limit your usual social activities with family or friends?	3.61	0.94
	H 7. During the past 4 weeks, how much have you been bothered by emotional problems (such as feeling anxious, depressed, or irritable)?	3.56	0.93
	H 8. During the past 4 weeks, how much did personal or emotional problems keep you from doing your usual work, school or other daily activities?	3.70	0.92
Quality of health care (QHC) (1–13)	QHC 1. Perform surgeries.	3.44	1.00
	QHC 2. Assess diagnostic information.	3.77	0.78
	QHC 3. Make correct diagnoses.	3.82	0.76
	QHC 4. Select appropriate treatments.	3.82	0.79
	QHC 5. Maintain medical records.	3.85	0.76
	QHC 6. Inform patients about rationale for treatment.	3.90	0.78
	QHC 7. Consider psychosocial aspects of illness.	3.77	0.79
	QHC 8. Manage health care resources efficiently.	3.82	0.78
	QHC 9. Evaluate medical literature to optimize clinical decision making.	3.73	0.83
	QHC 10. Participate in implementation of quality improvement programs.	3.73	0.82
	QHC 11. Show empathy for patients and their relatives.	3.93	0.78
	QHC 12. Involve patients in decision-making.	3.74	0.83
	QHC 13. Consider advance health care directives.	3.89	0.79

**Table 2:** Demographic characteristics of the final sample with information of the participants.

	Final Sample (*n* = 2426)	Percentage (%)
Sex		
Male	849	36.0
Female	1508	64.0
Age		
<25	225	9.4
25~30	679	28.4
31~35	568	23.8
36~40	344	14.4
41~45	219	9.2
46~50	196	8.2
>50	158	5.6
Education		
Below Junior College	121	5.1
Junior College	514	21.6
Bachelor Degree	1099	46.1
Master Degree	430	18.0
Doctor Degree	221	9.3
Title		
Primary	1058	45.6
Middle	844	36.4
Deputy Senior	297	12.8
Senior	119	5.1
Working Age (Year)		
<3	436	18.3
3~5	486	20.4
6~10	616	25.9
11~20	470	19.8
>20	369	15.5
Department		
Internal Medicine	582	24.7
Surgical	411	17.4
Maternity	248	10.5
Pediatric	207	8.8
Chinese Medicine/Rehabilitation	130	5.5
Emergency/ICU	128	5.4
Infection/Oncology	42	1.8
Other Clinical Department	142	6.0
Medical Technician	213	9.0
Administration and Logistics	108	4.6
Other	150	6.4

**Table 3:** Intercorrelations between Challenge Stress (CS), Hindrance Stress (HS), Quality of health care (QHC), and Health (H) items (** *p* < 0.01).

Variables (Mean (M), SD)	Items			
	CS	HS	QHC	H
CS (2.41, 1.39)	1			
HS (3.89, 0.66)	0.49 **	1		
QHC (3.66, 0.78)	0.08 **	−0.14 **	1	
H (3.66, 0.81)	−0.25 **	−0.36 **	0.25 **	1

**Table 4:** Standardized regression weights (β) with *p*-values (α = 0.05) for the components of the subgroup analyses.

	Female		Male		Young		Old		Primary		Secondary		Tertiary		Total	
	β	*p*	β	*p*	β	*p*	β	*p*	β	*p*	β	*p*	β	*p*	β	*p*
Path																
CS to H	−0.09	**	0.00	-	0.02	-	−0.11	**	−0.18	*	−0.03	-	−0.40	-	−0.06	**
CS to QHC	0.26	***	0.28	***	0.18	***	0.28	***	0.18	*	0.20	***	0.29	***	0.26	***
HS to H	−0.38	***	−0.41	***	−0.51	***	−0.31	***	−0.30	***	−0.34	***	−0.39	***	−0.37	***
HS to QHC	−0.24	***	−0.14	**	−0.31	***	−0.16	***	−0.20	*	−0.22	***	−0.17	***	−0.20	***
H to QHC	0.22	***	0.29	***	0.16	***	0.29	***	0.32	***	0.18	***	0.25	***	0.24	***

CS, challenge stress; HS, hindrance stress; H, health; QHC, quality of health care. * Significant at 0.01 < *p* < 0.05; ** Significant at 0.001 < *p* < 0.01; *** significant at *p* < 0.001. A dash (-) indicates that the regression weight was constrained to 1.0 in the proposed model.
